# Pazopanib-induced severe acute liver injury: A case report

**DOI:** 10.1097/MD.0000000000027731

**Published:** 2021-11-19

**Authors:** Jin-Wook Choi, Jeong-Ju Yoo, Sang Gyune Kim, Young Seok Kim, Susie Chin

**Affiliations:** a Division of Gastroenterology and Hepatology, Department of Internal Medicine, Soonchunhyang University Bucheon Hospital, Soonchunhyang University School of Medicine, Bucheon, Republic of Korea; b Department of Pathology, Soonchunhyang University Bucheon Hospital, Soonchunhyang University School of Medicine, Bucheon, Republic of Korea.

**Keywords:** acute cholestatic hepatitis, drug-induced liver injury, jaundice, pazopanib, renal cell carcinoma, toxic hepatitis

## Abstract

**Rationale:**

Drug-induced liver injury (DILI) is the most common cause of acute liver failure in the United States. Painkillers and fever antipyretics are the most common cause of DILI. Hepatic injury can be provoked by DILI as hepatocellular or cholestatic type.

**Patient concerns:**

A 48-year-old woman presented jaundice accompanied by nausea and vomiting. The patient was an inactive hepatitis B carrier with low viral titer and was diagnosed renal cell carcinoma (RCC) with hepatic metastasis requiring pazopanib treatment. Prior to administration of pazopanib, tenofovir administration was started to prevent exacerbation of hepatitis B. The patient was referred to clinic of gastroenterology department due to sudden elevation of bilirubin after 5 weeks of pazopanib treatment.

**Diagnoses:**

Abdominal ultrasound and computed tomography showed non-specific finding other than metastatic nodule in the liver and liver cirrhosis. After then, the patient was performed liver biopsy, and the biopsy result was acute cholestatic hepatitis with centrilobular area necrosis and portal inflammation. Therefore, considering the clinical history and biopsy results, the patient was diagnosed as DILI due to pazopanib.

**Interventions:**

After the biopsy, empirical steroid therapy was initiated and after 7 weeks of pazopanib discontinuation.

**Outcomes:**

The total bilirubin level returned to normal from peak level of 24.61 to 1.52 mg/dL.

**Lessons:**

In patients with renal cell carcinoma, pazopanib treatment requires clinical caution as it causes rare complications such as severe jaundice and acute cholestatic hepatitis.

## 1 Introduction

Drug-induced liver injury (DILI) causes about 10% of acute hepatitis and is emerging as the most common cause of acute liver failure in the United States.^[1]^ Typical causes in DILI are drugs, herbal medicines, and dietary supplements, especially when taking higher doses of painkillers and fever reducer than recommended.^[2]^

Pazopanib (Votrient; GlaxoSmithKline, Brentford, UK), the drug that was administered in this case, is an oral multi-targeted tyrosine kinase inhibitor and has been approved for metastatic renal cell carcinoma (RCC) and advanced soft tissue sarcoma.^[3]^ Tyrosine kinase inhibitors have been approved for treatment of metastatic RCC in Japan, the United States, and European countries.^[3]^ Pazopanib is metabolized in the liver, so impaired hepatic metabolism might have resulted in an increase of the serum concentration of pazopanib.^[4]^

Kidney cancer is the eighth most common cancer in the United States, and the majority of adult kidney neoplasms are RCC.^[5]^ Although the majority of patients present with localized disease, 20% of patients present with advanced disease, and 20% to 30% of all patients subjected to a nephrectomy with curative intent develop distant recurrence.^[6]^

This case report presents a 48-year-old Korean female patient with jaundice followed by 5-weeks of pazopanib treatment for RCC. Investigation and management approach is shared in the report and review of previous similar cases is provided as table.

## 2 Case presentation

A 48-year-old woman visited the referral university hospital to jaundice and nausea. She was referred to the outpatient clinic of gastroenterology department in a tertiary hospital due to jaundice and increased levels of liver enzyme detected during blood test tracking after 5 weeks of pazopanib therapy.

The patient's medical history included end-stage renal disease on hemodialysis, liver cirrhosis with hepatitis B virus carrier. The patient underwent right nephrectomy for RCC 2 years ago. Newly onset liver and lung metastasis were detected 3 months ago by liver biopsy. The patient started taking immunotherapy agent (pazopanib) and prophylactic antiviral agent (tenofovir disoproxil fumarate) from 2 months ago. Pazopanib prescribe was started at half dose considering the patient's clinical history of liver cirrhosis. After administration at 400 mg per day, which is half the dose for 2 weeks, liver enzyme level was kept in the normal range and showed no change, so the dosage was increased to a fixed amount of 800 mg per day and dosing was maintained for an additional 2 weeks.

Before first visiting the outpatient clinic, the patient had symptoms of nausea with vomiting and jaundice for 2 weeks. The patient was suspected to progress acute hepatitis considering jaundice and increased liver enzyme. Elevated serum total bilirubin persisted 2 weeks after pazopanib discontinuation and she was hospitalized for further evaluation and treatment.

At the time of admission, she was 160.5 cm tall, weighed 61.1 kg, and had a body mass index (BMI) of 23.72. Blood pressure of the patient was normal with a systolic blood pressure of 123 mm Hg and a diastolic blood pressure of 74 mm Hg. Mild elevated body temperature (37.8 °C) and normal heart rate (95 bpm) with normal breathing rate (18 breaths per minute) was seen. The patient presented with nausea and jaundice. The patient had a soft abdomen on physical examination.

**Figure 1:**
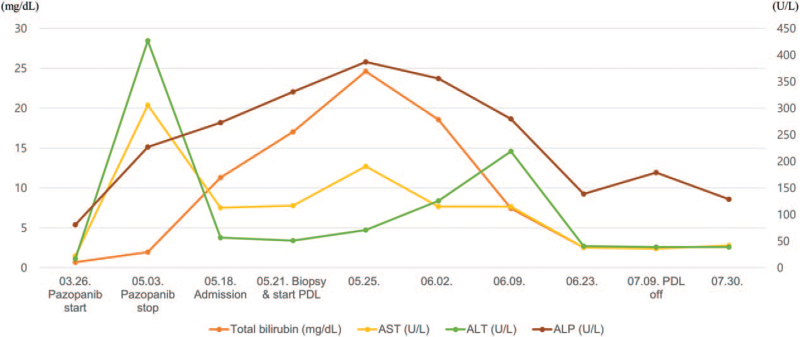
Clinical course of the patient. The patient had progressive jaundice, nausea, and vomiting 3 weeks after taking pazopanib. Therefore, the patient visited the outpatient clinic 5 weeks after pazopanib administration, and blood tests showed that total bilirubin and liver enzyme were elevated, and pazopanib administration was stopped. The patient was hospitalized because the bilirubin elevation did not improve even after 2 weeks of discontinuation of the drug, and liver biopsy was performed. The patient started prednisolone treatment for 7 weeks for pazopanib-induced DILI, and the improvement state was maintained even after PDL discontinuation. ALP = alkaline phosphatase, ALT = alanine aminotransferase, AST = aspartate aminotransferase, PDL = prednisolone.

Liver-function test at baseline shows no abnormal findings measured before pazopanib treatment and 2 weeks after the start of treatment. However, blood tests measured 5 weeks after the start of treatment revealed abnormal findings in liver function. At the time 5 weeks after starting treatment of pazopanib, levels of aspartate aminotransferase (AST), alanine aminotransferase (ALT), and alkaline phosphatase (ALP) were 306, 427, and 227 U/L, respectively. Prothrombin time-international normalized ratio value was in normal range (0.99). The total bilirubin level was 1.98 mg/dL (Fig. 1). Alpha-fetoprotein (AFP) level was 6.1 ng/mL with in normal range. The blood test results related to hepatitis virus infection are as follows, hepatitis B surface antigen (positive); anti-HBs (negative); hepatitis B e antigen (negative); anti-HBe (positive); hepatitis B virus DNA (120 copies/mL); anti-hepatitis C virus (negative). Child-Pugh score and class were 5 points and class A. The model for end-stage liver disease (MELD) score was 20 points and estimated 3-month mortality was 19.6%.

**Figure 2:**
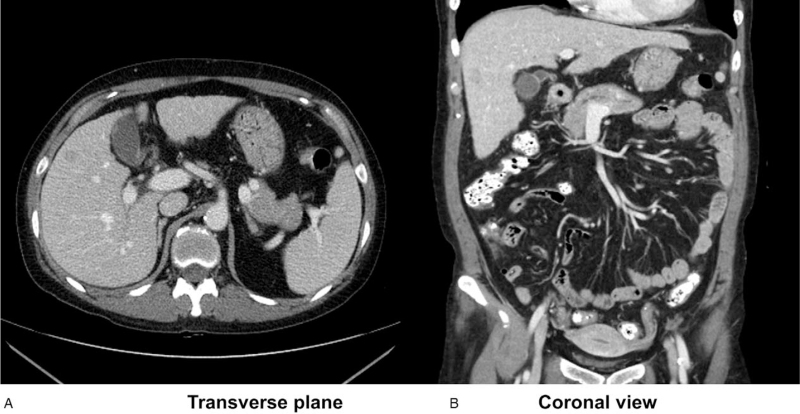
Computed tomography (CT) of the patient. (A) Transverse plane, (B) coronal view. Contrast-enhanced abdominal CT revealed liver cirrhosis and there was no significant change of about 1.3 cm sized enhancing hepatic nodule on right hepatic lobe which is pathologically proven metastatic renal cell carcinoma. There was no obstructive lesion on biliary tract.

Contrast-enhanced abdominal computed tomography (CT) revealed liver cirrhosis and there was no significant change of about 1.3 cm sized enhancing hepatic nodule on right lobe which is pathologically proven metastatic RCC. There was no obstructive lesion on biliary tract (Fig. 2). Abdominal ultrasound also revealed no significant abnormality other than hepatic nodule and liver cirrhosis.

There was a clinical possibility of DILI caused by pazopanib, and the Roussel Uclaf Causality Assessment Method (RUCAM) score was used to evaluate the possibility of the disease. The patient was of the mixed type. Symptoms started appearing 5 weeks after administration (+2). Total bilirubin, AST, and ALT were normalized within 180 days of the cessation of drug administration (+2). There were no risk factors such as alcohol, pregnancy, and the elderly over 55 years of age (0). Although tenofovir was taken as a concomitant drug, the possibility of hepatotoxicity by the drug was low, and the time interval was not consistent with the onset of symptoms (0). There were no acute viral hepatitis, bile duct obstruction, alcoholism, or recent hypotension that could cause liver damage other than drugs (+2). Pazopanib, which was taken, is a drug with a warning of hepatotoxicity on the product according to previous studies (+2). The possibility of DILI can be known more clearly through re-administration of the drug, but re-administration was not performed in this patient (0). Taking these factors into consideration, the patient's RUCAM score was 8 points, and it was in the probable group corresponding to 6 to 8 points, so the possibility of DILI was high. At the beginning of the clinical course, it was unknown whether there will be any improvement after the cessation of drug administration, so even if this score factor (course-change after stopping drug) was excluded, the RUCAM score was already 6 points, indicating a high possibility of DILI, so treatment for pazopanib-induced DILI was initiated.

**Figure 3:**
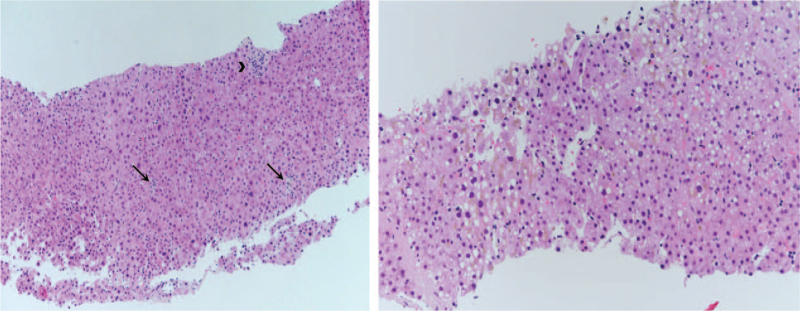
Results of liver biopsy. (A) Histology showed scattered foci of spotty necrosis (arrow), hepatocellular cholestasis, and hepatocyte disarray. The protal tract showed only sparse chronic inflammation (arrow head) (Hematoxylin and eosin staining [H&E], ×100). (B) There is hepatocyte swelling and degeneration with cholestasis in centrilobular area (H&E, ×200).

Finally, for confirmatory diagnosis, liver biopsy was performed to establish the cause of jaundice. Liver biopsy revealed hepatocyte swelling and degeneration with cholestasis in centrilobular area and the protal tract showed only sparse chronic inflammation. The final diagnosis was confirmed as acute cholestatic hepatitis consistent with toxic hepatitis (Fig. 3). Therefore, the patient was diagnosed with severe DILI due to pazopanib.

The patient started to take prednisolone 40 mg daily for 1 week for empirical treatment of DILI. The dose of prednisolone was gradually reduced every 1 week and it was discontinued finally after 7 weeks of dosing. After discontinuation of pazopanib and taking empirical steroid treatment, the patient's AST and ALT levels were maintained within the normal range and total bilirubin level was decreased significantly from the peak level of 24.61 to 1.06 mg/dL on 12-weeks after pazopanib discontinuation (Fig. 1). After discontinuation of pazopanib treatment, jaundice improved rapidly and the patient is currently being observed in the outpatient clinic.

The study protocol was approved by the Institutional Review Board of Soonchunhyang University Bucheon Hospital, and conformed to the ethical guidelines of the World Medical Association Declaration of Helsinki. Written informed consent was obtained because of the retrospective design.

## 3 Discussion

Pazopanib which has been prescribed for metastatic RCC and advanced soft tissue sarcoma was approved with a black box warning about hepatotoxicity. In the phase III trial, the incidence of grade 3 to 4 ALT increase was 12%, and 53% of patients experienced some degree of hypertransaminasemia.^[7]^ The incidence of ALT elevation in pazopanib ranges from 46% to 60% in other study.^[8]^ A subsequent meta-analysis of all pazopanib-containing trials confirmed a 42% incidence of all-grade ALT increase and an 8.2% incidence of high-grade ALT increase to above 5 times upper normal limit.^[9]^ In preliminary trials of pazopanib in various solid tumors, there were rare reports of hepatitis with jaundice in <1% of patients. In this report, demographic and clinical characteristics of the 6 cases are presented in Table 1. The clinical onset is typically within 5 to 6 weeks of starting pazopanib and the recovery period varied from 3 to 10 weeks after stopping pazopanib. Our patient also showed markedly elevated ALT and ALP levels with jaundice after treatment with pazopanib. In our patient, ALP levels increased 5 weeks after the initiation of pazopanib therapy.

**Table 1:** Characteristics of pazopanib-induced liver injury based on prior case reports.

		Demographic and clinical characteristics				Course of illness				Initial values			
References	Year	Location	Sex	Age, yr	Daily dose, mg	Symptoms	Latency, wks	Outcome	Baseline liver disease	ALT, U/L [0–35]	ALP, U/L [30–120]	Total bilirubin, mg/dL [0.2–1.2]	INR [0.87–1.13]
Klempner et al^[10]^	2012	USA	M	77	800	Jaundice	6	Death	None	759	729	6.3	NA
Klempner et al^[10]^	2012	USA	M	62	800	Jaundice	6	Recovery after 5 weeks	None	114	89	0.5	1
Hayashi et al^[11]^	2021	Japan	M	72	800	Jaundice	4	Recovery after 10 weeks	None	487	1127	2.9	0.99
Kuribayashi et al^[12]^	2017	Japan	F	68	600	Fatigue, palpitation	3	Recovery after 3 weeks	None	953	NA	NA	NA
Verschoor et al^[13]^	2018	Netherlands	F	40	800	None	6	Recovery after 6 weeks	None	× 10 UNL	NA	Normal	NA
Our case	2021	Republic of Korea	M	48	800	Jaundice, nausea	5	Recovery after 7 weeks	HBV carrier	427	227	1.98	0.99

Normal reference ranges were showed as follows []. ALP = alkaline phosphatase, ALT = alanine aminotransferase, F = female, HBV = hepatitis B virus, INR = international normalized ratio, M = male, NA = not applicable.

The mechanism of injury accounting for serum enzyme elevations during pazopanib therapy is not known. Pazopanib is metabolized in the liver largely through the cytochrome P450 3A4 pathway and liver injury may be related to production of a toxic intermediate. Pazopanib is susceptible to drug–drug interactions with agents that inhibit or induce hepatic cytochrome P450 3A4 activity.^[14]^ Retrospective analyses have suggested that other medications likes simvastatin may play a role in serum aminotransferase elevations. Recently, an association between uridine diphosphate glucuronosyltransferase 1A1 (UGT1A1) polymorphisms and pazopanib-induced liver toxicity was observed.^[15]^ The UGT1A1 enzyme is inhibited by pazopanib, and patients carrying the UGT1A1∗28 allele (also known as TA7, where TA6 represents the wild type allele) have a risk of hyperbilirubinemia. If acute liver injury with no other cause occurs during pazopanib administration, the presence of UGT1A1 gene polymorphisms should be checked. UGT1A1 assessment could improve the management of pazopanib-induced liver toxicity in patients with metastatic RCC. More recently, an association between human leukocyte antigen B∗57:01 and pazopanib-induced liver toxicity was also found.^[16]^

Pazopanib is the drug of choice for patients with advanced RCC and sarcoma. However, pazopanib-induced DILI can be fatal and it can lead to discontinuation of treatment and even chronic damage to liver function. In the case of toxic hepatitis caused by pazopanib administration, liver damage can be improved by administration of prednisolone for treatment. In other cases investigated as references in this case report and the patient in this report, prednisolone was administered and transaminase and total bilirubin level were decreased. Generally, the history of old age, high BMI, prolonged PT, and cholestatic patterns of liver injury can be sign of severe liver injury associated with the high risk of jaundice and elevated serum transaminase. There are not any well-controlled studies to corroborate these findings, but these can be high risk factors for pazopanib-induced liver injury.

Most of patients resolves with immediate discontinuation of the drug, but DILI can be worsen and progress to failure of survival, requiring liver transplantation or even death, especially if drug discontinuation is delayed. Patients receiving pazopanib treatment should be accompanied by baseline liver function tests and routine monitoring during the dosing period for every 3 weeks. If serum aminotransferase elevation >5 times the upper normal limit or any elevation with jaundice or symptoms is confirmed, patients should be leaded to dose reduction or temporary cessation.

## Author contributions

**Approval of final manuscript:** all authors.

**Conceptualization:** Jeong-Ju Yoo.

**Data curation:** Jin-Wook Choi.

**Formal analysis:** Jin-Wook Choi.

**Investigation:** Jeong-Ju Yoo.

**Methodology:** Sang Gyune Kim.

**Pathological findings description:** Susie Chin.

**Project administration:** Jeong-Ju Yoo.

**Resources:** Jin-Wook Choi.

**Supervision:** Young Seok Kim.

**Visualization:** Jin-Wook Choi.

**Writing – original draft:** Jin-Wook Choi.

**Writing – review & editing:** Jeong-Ju Yoo.

## References

[1] Ghabril M Chalasani N Björnsson E. Drug-induced liver injury: a clinical update. Curr Opin Gastroenterol 2010;26:222–6. 20186054 10.1097/MOG.0b013e3283383c7c PMC3156474

[2] Fontana RJ Seeff LB Andrade RJ. Standardization of nomenclature and causality assessment in drug-induced liver injury: summary of a clinical research workshop. Hepatology 2010;52:730–42. 20564754 10.1002/hep.23696 PMC3616501

[3] Davidson BA Secord AA. Profile of pazopanib and its potential in the treatment of epithelial ovarian cancer. Int J Womens Health 2014;6:289–300. 24648773 10.2147/IJWH.S49781 PMC3958497

[4] van Geel RM Beijnen JH Schellens JH. Concise drug review: pazopanib and axitinib. Oncologist 2012;17:1081–9. 22733795 10.1634/theoncologist.2012-0055 PMC3425526

[5] National Cancer Institute. Cancer Stat Facts: Kidney and Renal Pelvis Cancer. Available at: https://seer.cancer.gov/statfacts/html/kidrp.html. Accessed August 1, 2021.

[6] Hsieh JJ Purdue MP Signoretti S. Renal cell carcinoma. Nat Rev Dis Primers 2017;3:17009. 28276433 10.1038/nrdp.2017.9 PMC5936048

[7] Sternberg CN Davis ID Mardiak J. Pazopanib in locally advanced or metastatic renal cell carcinoma: results of a randomized phase III trial. J Clin Oncol 2010;28:1061–8. 20100962 10.1200/JCO.2009.23.9764

[8] Powles T Bracarda S Chen M. Characterisation of liver chemistry abnormalities associated with pazopanib monotherapy: a systematic review and meta-analysis of clinical trials in advanced cancer patients. Eur J Cancer 2015;51:1293–302. 25899987 10.1016/j.ejca.2015.03.019 PMC7451810

[9] Kapadia S Hapani S Choueiri TK Wu S. Risk of liver toxicity with the angiogenesis inhibitor pazopanib in cancer patients. Acta Oncol 2013;52:1202–12. 23594201 10.3109/0284186X.2013.782103

[10] Klempner SJ Choueiri TK Yee E Doyle LA Schuppan D Atkins MB. Severe pazopanib-induced hepatotoxicity: clinical and histologic course in two patients. J Clin Oncol 2012;30:e264–8. 22802316 10.1200/JCO.2011.41.0332

[11] Hayashi M Abe K Fujita M. Pazopanib-induced mixed liver injury in a patient with soft-tissue sarcoma, but without the UGT1A1∗28 mutation: a case report. Clin J Gastroenterol 2021;14:224–8. 32990937 10.1007/s12328-020-01253-x

[12] Kuribayashi S Takao T Okuda Y. Case of pazopanib-induced thyrotoxicosis in a patient with metastatic renal cell carcinoma. Int Cancer Conf J 2017;6:118–20. 31149484 10.1007/s13691-017-0288-8 PMC6498272

[13] Verschoor AJ Warmerdam F Bosse T Bovée J Gelderblom H. A remarkable response to pazopanib, despite recurrent liver toxicity, in a patient with a high grade endometrial stromal sarcoma, a case report. BMC Cancer 2018;18:92. 29357824 10.1186/s12885-018-3999-0 PMC5778698

[14] Vincenzi B Armento G Spalato Ceruso M. Drug-induced hepatotoxicity in cancer patients - implication for treatment. Expert Opin Drug Saf 2016;15:1219–38. 27232067 10.1080/14740338.2016.1194824

[15] Henriksen JN Bøttger P Hermansen CK. Pazopanib-induced liver toxicity in patients with metastatic renal cell carcinoma: effect of UGT1A1 polymorphism on pazopanib dose reduction, safety, and patient outcomes. Clin Genitourin Cancer 2020;18:62.e2–8.e2. 31640912 10.1016/j.clgc.2019.09.013

[16] Xu CF Johnson T Wang X. HLA-B∗57:01 confers susceptibility to pazopanib-associated liver injury in patients with cancer. Clin Cancer Res 2016;22:1371–7. 26546620 10.1158/1078-0432.CCR-15-2044 PMC7444994

